# Rehabilitation Treatment of Motor Dysfunction Patients Based on Deep Learning Brain–Computer Interface Technology

**DOI:** 10.3389/fnins.2020.595084

**Published:** 2020-10-22

**Authors:** Huihai Wang, Qinglun Su, Zhenzhuang Yan, Fei Lu, Qin Zhao, Zhen Liu, Fang Zhou

**Affiliations:** Department of Rehabilitation Medicine, The First People’s Hospital of Lianyungang, Lianyungang, China

**Keywords:** rehabilitation treatment, motor dysfunction patients, deep learning, brain–computer interface technology, EEG

## Abstract

In recent years, brain–computer interface (BCI) is expected to solve the physiological and psychological needs of patients with motor dysfunction with great individual differences. However, the classification method based on feature extraction requires a lot of prior knowledge when extracting data features and lacks a good measurement standard, which makes the development of BCI. In particular, the development of a multi-classification brain–computer interface is facing a bottleneck. To avoid the blindness and complexity of electroencephalogram (EEG) feature extraction, the deep learning method is applied to the automatic feature extraction of EEG signals. It is necessary to design a classification model with strong robustness and high accuracy for EEG signals. Based on the research and implementation of a BCI system based on a convolutional neural network, this article aims to design a brain–computer interface system that can automatically extract features of EEG signals and classify EEG signals accurately. It can avoid the blindness and time-consuming problems caused by the machine learning method based on feature extraction of EEG data due to the lack of a large amount of prior knowledge.

## Introduction

**FIGURE 1:**
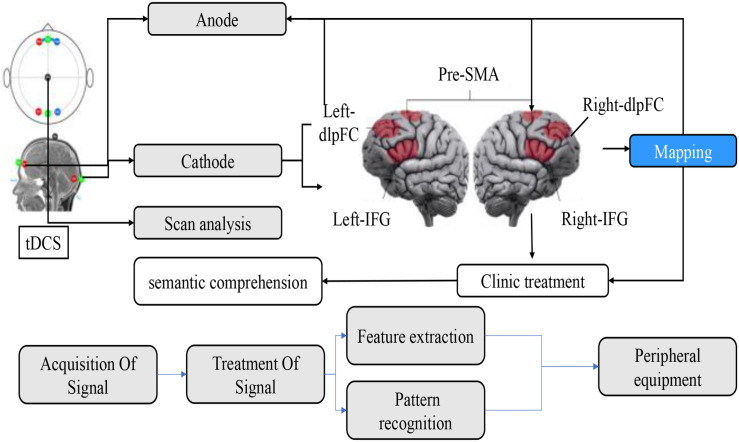
Schematic diagram of brain information processing.

Brain–computer interface (BCI) is a communication control system established between the brain and external devices (computers or other electronic devices) through signals generated by brain activity. The system does not depend on muscles and nerves other than the brain and establishes direct communication between the brain and the machine (Wang et al., 2020). It is a new, high-end way of human–computer interaction. The complete BCI system includes four parts: signal acquisition part, the feature extraction part, pattern recognition part, and control command output part (Turgut et al., 2018). The brain’s thinking activity mainly depends on the central nervous system. When human beings carry out different thinking activities, the neural activity patterns in the brain are altered, and the signals generated by the neural activity are different. In theory, the BCI system can monitor the signals generated by neural activities through a variety of sensors and other signal acquisition equipment. Through the analysis and processing of the signals, that is, feature extraction and pattern recognition in the following section, the signals are classified according to separate thinking activities to generate corresponding control commands to complete the interactive tasks between users and external devices. Feedback is not necessary. It is usually used in online BCI systems so that users can see their thinking corresponding to the control results as shown in Figure 1. If the consequences are different from expectations, users can adjust their thinking in time and have a better user experience.

The activities of the nervous system in the brain are very complex, including electrophysiological activities, neurochemical reactions, and metabolic phenomena in the central nervous system, such as action potential, postsynaptic potential, neurotransmitter release, oxygen metabolism, and so on. During this period, there are a variety of signals, including electrical signals, magnetic signals, chemical signals, and so on. According to the way of signal acquisition, the BCI system can be divided into two categories: intrusive and non-invasive (Arndt et al., 2017). For the detection of chemical signals, such as the changes of oxygenated hemoglobin or other physiological parameters, it is generally necessary to use an invasive BCI system, that is, to record signals in the brain by implanting sensors into the skull. For magnetic signal detection, magnetoencephalography (MEG) (Zesiewicz et al., 2018) can be utilized, which is a non-invasive device, but it has the advantages of large size, high price, and low time resolution. For the detection of electrical signals, there are two commonly used methods: electrocorticogram (electrocorticography) and electroencephalogram (EEG). Among them, electrocorticogram is an invasive acquisition method (Earnshaw et al., 2019; Levy et al., 2019; Tian et al., 2019), which extracts electrical signals from the cortex by implanting a microelectrode array into the cerebral cortex. The amplitude of the signal extracted by this method is large and the signal-to-noise ratio is high, but because of the craniotomy. The risk coefficient is high. EEG is a non-invasive acquisition method, which detects weak EEG signals by placing electrodes on the scalp, filters and amplifies them, and records the changes of scalp electrical signals during brain nerve activity. The equipment is light and portable, the price is relatively low and easy to operate, but because the process of EEG transmission from the cerebral cortex to the scalp will be greatly weakened, the signal-to-noise ratio of the extracted signal is very low, which increases the difficulty of subsequent feature extraction. At present, the most studied BCI system is the one which collects scalp EEG as the source signal in a non-invasive way. With the continuous maturity of BCI, the BCI system will gradually come into people’s everyday life. Considering multiple factors such as dangerous, maneuverability, price, and so on, collecting EEG through wearable equipment will become the main development direction of this technology and get the most extensive application.

Although human beings have mastered some knowledge about medicine, physiology, and pathology of the brain, for such a complex brain, these are only the tip of the iceberg; anthropological understanding of the brain is not very clear. Through the study of BCI and the analysis of the signals generated by the human brain, it can indirectly help human beings to explore the brain and understand the structure, working principle, and cognitive law of the brain. The knowledge obtained can not only promote the study of the brain–computer interface in turn but also be combined with other scientific directions such as computer and chemistry to promote the development of related disciplines. For example, the law of human brain vision cognition obtained through the study of vision-evoked brain–computer interface has inspired researchers to improve computer vision models and greatly promoted the research progress in the field of machine vision. CI technology is widely used in the medical field, mainly focused on the research and development of medical rehabilitation aid. For patients with epilepsy, a medical device can be designed to identify abnormal brain activity (Shulman et al., 2020). When abnormal brain activity is detected, a pulse signal is directed to destroy the prerequisite for seizures. For patients with insomnia, wearable devices can be developed to improve sleep quality (Drew et al., 2017), to understand the current sleep state by detecting EEG and to introduce auditory stimulation to guide patients into a deep sleep. For people with muscle paralysis, devices such as wheelchairs or robotic arms controlled by EEG signals can be improved to help people with motor disabilities complete movements that they cannot achieve and improve their ability to take care of themselves. Based on the research and implementation of a brain–computer interface system based on a convolutional neural network, this article aims to design a brain–computer interface system that can automatically extract features of EEG signals and classify EEG signals accurately. It can avoid the blindness and time-consuming problems caused by the machine learning method based on feature extraction in feature extraction of EEG data due to the lack of a large amount of prior knowledge. The purpose of this article is to explore the extension of the intense learning method to the field of brain–computer interface. The classification and recognition of EEG signals are the core focus of the development of BCI. Without the classification of EEG signals, the development of BCI is impossible. Therefore, to explore how to use the innate learning method to design an efficient classification model for the classification and recognition of EEG signals under the new situation is helpful to promote the development of BCI (Jiang et al., 2017). At the same time, exploring the classification method of EEG signals based on a multilayered neural network plays an important role in promoting the practicality of the brain–computer interface. In this article, the left-handed and right-handed motor imagination EEG classification model based on convolution neural network developed by Google machine learning framework contraflow achieves 75.3% classification accuracy on the test set of BCI Competition IV common data set, Data set 2b, and it can be transplanted to mobile phones, computers, tablets, and other terminal devices, used in brain–computer interface technology, in medical rehabilitation in the field of healthcare. For some, physical disabilities but normal brain function, such as muscle atrophy, spinal injury, and limb paralysis, provide a certain degree of convenience in life.

The rest is organized as follows. In Section 2, we review some related works. In Section 3, we state our data and method. In Section 4, we show the experimental results and discuss the results. Lastly, we conclude our study and indicate our future studies.

## Related Work

### Classification of EEG Signals Based on Machine Learning Method of Feature Extraction

In 1990, Kole proposed the common space template (CSP) method based on the eigenfunction method of Hjorth and Rodin. Kole uses the CSP method to process EEG data, which can distinguish whether the tested object is a patient or not. The accuracy of the test results is between 71% and 85%. Based on Kole’s proposal, Gerking, Herbert, and Ramoser put forward the method of solving the optimal value of the spatial template (Ferragut-Garcías et al., 2017). In 2010, Subasi used a new classification method based on his original research results, absorbed the support vector machine (SVM) theory, and introduced independent principal component analysis (ICA), principal component analysis (PCA) and other methods in feature extraction. The accuracy of EEG classification has been improved to more than 98%. In 2011, Deepa used a combination of instance-based (IB1) and alternating decision tree (tree) to classify human EEG signals, using EEG energy data calculated by GS-FHT method, and Chebyshev filter was used for initial signal processing. Deepa confirmed that using the GS-FHT method is 3–4% points more accurate than the FHT method used. Wu Linshang and others have done a lot of research in this area, which shows that in extracting the features of the data, through the CSP (ordinary spatial pattern) method, when classifying the data, the LDA (linear recognition analysis) method is used to get about 80% of the 2 classification results of the motion imagination data. Huang uses the feature extraction methods of surface Laplace transform (SLD) and power spectral density (PSD), combined with the classification method of support vector machine (SVM) to recognize motion imagination EEG data, and applies its results to the control of the two-dimensional mouse. We used a common spatial pattern (CSP) for feature extraction, then used linear discriminant (LDA) for motion imagination data classification, and achieved good results.

### Classification of EEG Signals Based on Deep Learning Method

In recent years, with the arrival of the big data era and the popularity of GPU parallel computing, deep learning has received unprecedented attention, and its related theories have been continuously improved and developed. Nowadays, the model built by deep learning method has won the championship in many competitions between pattern recognition and machine learning and has brought breakthroughs in the fields of image recognition, speech recognition, and natural language processing. Deep learning is developed from machine learning and is very good at automatic feature extraction from complex unstructured data. In the early days of artificial intelligence, traditional algorithms such as logical regression and Bayesian classification already have the ability of machine learning, but these algorithms do not learn from the original data but require manual feature representation of the input data. For example, for the problem that the input is X and the expected output is Y, the researchers should first design the Feature (X), and then learn the mapping F from the traditional algorithm to make the Y-shaped feature F. The disadvantages of this kind of methods are obvious, and how to extract the features effectively has become a new problem to be solved. On this basis, representation learning arises at the historic moment (Pin-Yeh and Qiang, 2017). For the problem that the input is X and the expected output is Y, the mapping function is constructed, and the features that need to be extracted manually are also automatically learned by the algorithm through machine learning. Self-encoder belongs to this kind of algorithm, this kind of calculation not only saves manual time and energy, but also the features obtained often have better expression performance than those selected by hand. For some very complex problems, the shallow features obtained by learning can no longer meet the needs of the problem, so deep learning begins to develop. This kind of method constructs complex features through simple features, abstracts them step by step, and finally extracts the high-level features that can solve the problem through multi-layer representation, that is, the process of solving the problem. Although the increase of depth makes the deep learning algorithm have more powerful feature representation ability, it also makes the process of training optimization easily fall into the local extremum and produce the phenomenon of gradient disappearance, and the effect is not ideal. BP network is an example. Later, Hinton and others put forward the concept of deep belief network in 2006, successfully introduced the pre-training process and fine-tuning technology, and used greedy strategy to train the deep architecture composed of restricted Boltzmann machine layer by layer (Barfod et al., 2020; Smith et al., 2020), which not only solved the convergence problem of the depth model but also greatly reduced the time needed for deep network training, which started the wave of deep learning. Nowadays, researchers all over the world have done a lot of research in this field, and deep learning algorithms have been greatly expanded and developed, including convolutional neural networks, cyclic neural networks, stacked self-coding networks, and deep belief networks. Deep Boltzmann machines have made breakthroughs and have been widely used in different fields. Combined with the characteristics of EEG signals, this article chooses a convolution neural network (convolutional neural networks, CNN) and long-term short-term memory circulation neural network (long short-term memory networks, LSTM) for in-depth research. The following article will focus on the principle, advantages, and reasons for choosing this algorithm.

**FIGURE 2:**
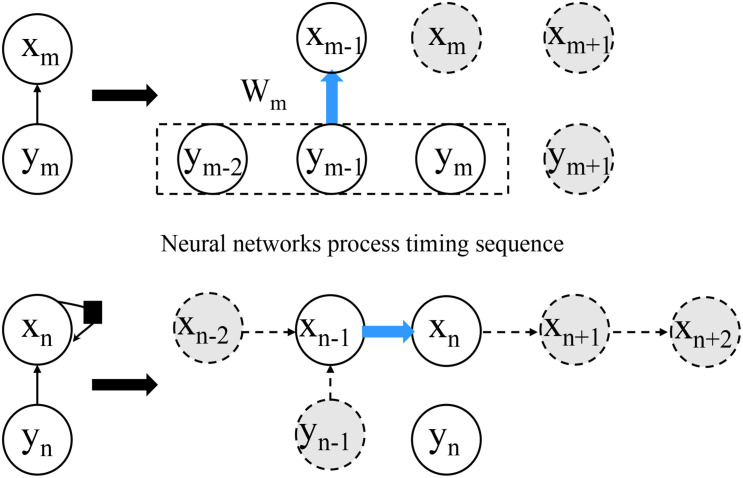
Distributed system for neural network process timing sequence.

The convolution neural network model is the first neural network based on the concept of the local receptive field constructed by Fukushima in 1984. This model is inspired by the process of human visual abstraction and is widely used in image and video recognition. In the 2012 Image Net competition, previous work won the competition by relying on the five-layer convolution network model Alex Net constructed by the convolution neural network algorithm and achieved a good result of 1000 classification error rate of 100000 images with an error rate of 15.3% (Zhang et al., 2017). In recent years, through the continuous efforts of a large number of researchers and improving the 152-layer Res Net model designed by He K and others, the error rate has been reduced to 3.6% (Kubota et al., 2019), which makes the image recognition ability of machines surpass that of human beings. In Telles et al. (2019), Deng et al. used CNN to extract different kinds of features from EEG signals. A complete convolution neural network model usually consists of an input layer (picture), multiple alternating convolution layers and pooling layers, a full connection layer, and a final output layer. And the distributed system for the neural network process timing sequence can be seen in Figure 2.

**FIGURE 3:**
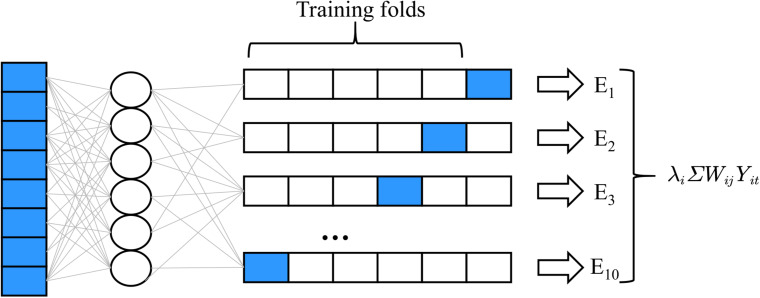
The structure of a neuron in the neural network model.

The structure of a neuron in the neural network model is shown in Figure 3. The first difference between the convolution neural network and the traditional neural network lies in the choice of the activation function σ. In the traditional neural network, σ is usually selected as the sigmoid function or tanh function, while the activation function σ of the convolution neural network is the real function.

Additionally, some deep ensemble methods are also used for EEG classification. For example, Zhang et al. (2019d) proposed a deep view-reduction TSK fuzzy system for epileptic EEG signals recognition. It is a deep ensemble method in which 0-order TSK fuzzy systems are linked in a stacked manner so as to improve the classification performance.

### Research on Visual Driving Potential System

**FIGURE 4:**
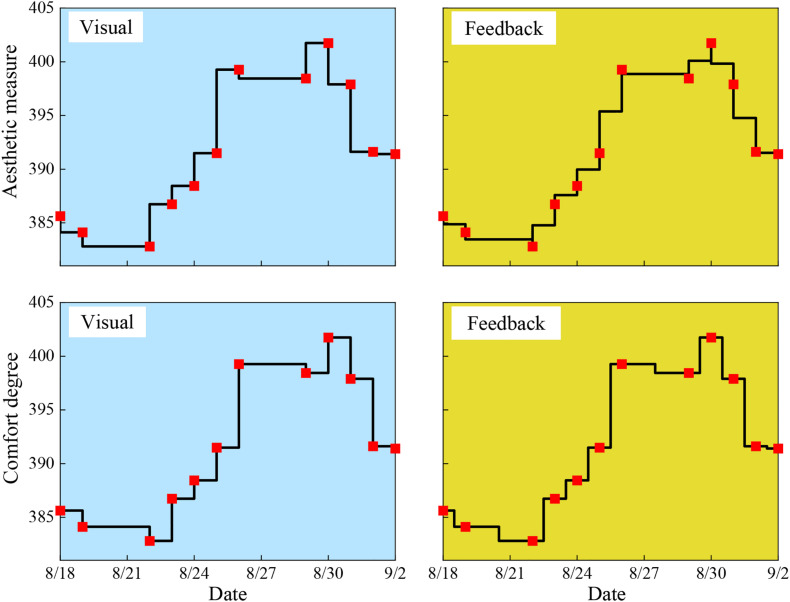
The visual and feedback signals for different dates.

Visual evoked potential (VEP), also known as visual evoked potential (VEP), is one of the evoked BCI EEG signals. Its principle is to produce measurable potential changes in the visual cortex through visual stimulation (flicker stimulation, change of stimulus brightness, color alternation, etc.). According to the frequency of stimulation, VEP can be divided into transient evoked potential (transient visual evoked potential, TVEP) and steady-state evoked potential (steady state visually evoked potentials, SSVEP). The frequency of TVEP stimulation is low, and the next stimulation will not be carried out until the response produced by a single stimulation in the cerebral cortex disappears, while the stimulation frequency of SSVEP is high, and the next superimposed stimulation will be carried out directly before the response produced by a single stimulation in the cerebral cortex has disappeared (Bober et al., 2018; Whyte et al., 2019). In the BCI system with VEP as the paradigm, there are more researches on the use of SSVEP stimulation, and the system of this paradigm is also the fastest among the non-invasive BCI systems at present. American Calhoun G.L. Using steady-state visual evoked potentials to complete the task of brainwave control to simulate aircraft (Jiang et al., 2017; Zhang et al., 2019b, d) the BCI system based on the SSVEP paradigm developed by, previous work realized the functions of the mind control switch, mobile dialing and so on (Lopes et al., 2017). Because the stimuli and visual evoked potentials have the same rhythm, it is easier to determine the subjects’ gaze commands, the EEG signals generated by this paradigm have good stability, high signal-to-noise ratio, and a large number of identifiable classifications. However, this paradigm requires subjects to look at the screen for a long time, which is easy to feel tired, and the scheme needs to select targets with the help of the subjects’ eye movements, so it cannot be applied to blind and completely paralyzed patients. The evoked potentials generated based on the SSVEP paradigm were distributed in the occipital lobe of the scalp, the frequency was between 1 Hz and 300 Hz, and the duration was about 200 ms (Skolasky et al., 2020). The evoked potentials were recorded with 1 to 8 electrodes. The EEG power spectrum analysis method commonly used in the single-lead acquisition is (Power Spectral Density, PSD) to extract frequency domain features (Zhang et al., 2018, 2019a, 2020a; Zhao et al., 2019; Zheng et al., 2019), and the typical correlation analysis method (canonical correlation analysis, CCA) in multi-lead acquisition EEG is commonly used to extract spatial features (Pereira et al., 2019). Before feature extraction, it is necessary to filter and fuse the original EEG signal to improve the signal-to-noise ratio (Stan et al., 2019). We use wavelet denoising for preprocessing, and the processing process of pattern classification by CCA achieves 85% accuracy in four classifications. Literature (Dong et al., 2017) adds the processing step of channel selection, which increases the accuracy to 87.5%. Literature (Bai and Fong, 2020) proposes a method of combining independent component analysis with Hilbert-Huang transform, which can effectively extract features but takes a long time. Literature (Schneider et al., 2017) uses principal component analysis to improve CCA to ensure accuracy and improve the speed of signal recognition at the same time. Previous work (Bober et al., 2020) uses a wavelet packet to improve CCA as shown in Figure 4, which improves accuracy by about 5% and gets 82% accuracy. In the literature, a power spectrum Gaussian method based on empirical mode decomposition is proposed to achieve 84% accuracy.

### Convolutional Network and Recurrent Neural Network

#### Convolution Network

The traditional fully connected network has many disadvantages. Taking the problem of image recognition as an example, if we take a picture of 1000 × 1000 pixels as the input of the network and set the number of neurons in the first hidden layer to 100, then only the first layer has 100 biases (b) and 100000000 weights (w) need to be trained, the number of parameters of the network is too many, the training is extremely difficult. On the other hand, the convolution network makes full use of the correlation difference of image pixels in different positions and adopts three methods: local receptive field, weight sharing, and downsampling, which provides an effective solution to the problem of image recognition. For the pixels in the picture, the relationship between the adjacent pixels is larger, and the relationship between the distant pixels is smaller, so it is not necessary to connect the neurons with each pixel in the input image. We only need to connect the local of the previous layer, scan and extract the features in blocks, and then synthesize the local features at a deeper level to get the global features. This process is very similar to the visual process. In the human visual system, each neuron corresponds to a part of the visual domain. In the convolution model, each convolution nucleus is only connected to the part of the neurons in the previous layer to extract a local feature. The connection between neurons through the local receptive field has reduced the parameters by a large part, and weight sharing can further reduce the number of parameters. Take the input picture of 1000 × 1000 pixel as an example, if the convolution kernel size of the first layer is 10 × 10, the meaning of weight sharing means that the weight of 10 × 10 on the convolution kernel is the same to the 1000 × 1000 input layer neurons, that is, there are only 100 parameters of the weight, and the weight trained in a certain location is still valid in other locations. In theory, the features extracted by the convolution kernel can be directly used in the training of the classifier, but the high dimension of the feature vector is not conducive to training, and it is easy to produce over-fitting phenomenon. The convolution network puts forward the process of adding downsampling after convolution, and aggregates and statistics the features of different locations. This process, also known as pooling, can not only reduce the dimension of features to improve training efficiency but also prevent overfitting. The specific pooling methods include maximum pooling, average pooling, and so on. The maximum pooling only retains the maximum value of the pooling window, while the average pooling retains the average value of the pooling window. Although the convolution neural network model is inspired by the visual abstraction process and has made brilliant achievements in the field of picture recognition and computer vision, however, this does not mean that convolution neural networks can only be applied to the recognition and processing of vision-related signals such as pictures and videos. Through the above analysis of the principle and method of the convolution neural network model, it is reasonable to think that the model can also be applied to the feature extraction and classification of EEG signals. Because the convolution neural network model is proposed for the problem of taking pictures as input, the structure of the model is very beneficial to the feature extraction and classification of two-dimensional or multi-dimensional matrix inputs. The EEG signal collected by the subject is two-dimensional matrix data, which satisfies the input form of a convolution neural network. The use of convolution network not only solves the training difficulties caused by the high dimension of EEG signals but also gives full play to the shape advantage that EEG signals are two-dimensional matrix (Zhang et al., 2016, 2019c, 2020b; Polkey et al., 2018). The structure of the network model is very clear. The convolution network uses the local receptive field to solve the problem of too many parameters of the network. The premise of this method is that the closer the pixel in the picture is, the larger the relationship is, and the farther the distance is, the smaller the relationship is. If the cerebral cortex is regarded as a picture, and the voltage collected by the electrode placed on the cortex is regarded as a pixel, then the EEG signal has a position relationship similar to that of the picture pixel. Therefore, the method of the local receptive field in the convolution network can be used to process EEG signals without losing features (Pavlova et al., 2017). The weight sharing strategy adopted by the convolution neural network makes the network translation invariant, which is shown in the picture, although the extracted feature position has changed after the translation operation of a picture. But the network can still correctly identify the picture. It is shown in the EEG that if the electrode cap used in the collection is not worn, there is a deviation in the distribution position of the conductive electrode, or due to the slow response of the subjects, the acquisition equipment is not sensitive and other factors such as time-shift (Kim et al., 2019) will not affect the classification results of EEG signals. This is consistent with the actual demand, so the weight sharing strategy in the convolution network is also suitable for EEG signal processing. The pooling layer in the convolution network reduces the feature dimension extracted by the convolution layer and aggregates the features in different positions of the image. According to the calculation principle of average pooling, it is known that this method makes the network have certain distortion invariance, that is, a certain amount of distortion operation on the input image will not affect the recognition of the image. The EEG signal has the characteristics of high noise and strong randomness. The motion imagination EEG signal of different people or even the same person at a different time is different every time. Pooling operation can filter noise and increase the robustness of the algorithm to a certain extent. To sum up, although EEG is a voltage signal and image is pixel information, they have many common characteristics, they can share local information, and the features and features are homogeneous. Also, the convolution neural network algorithm has the advantages of fast training speed and strong ability of spatial feature extraction, so the convolution neural network algorithm (CNN) is selected for an in-depth study of MI EEG signals.

#### Recurrent Neural Network

The recurrent neural network (RNN) model was proposed by Paul Werbos in 1988. To better deal with the sequence data, the concept of loop is added to the structure to persist the information. The unique loop structure of the algorithm enables the network to remember the previously input information and apply the useful information in the memory to the calculation of the subsequent output, which is very suitable for text, voice, and video. This model has made great achievements in many fields of time series analysis, such as speech recognition, natural language processing, machine translation, and so on. It is called the two most popular deep learning algorithms together with a convolution neural network.

**FIGURE 5:**
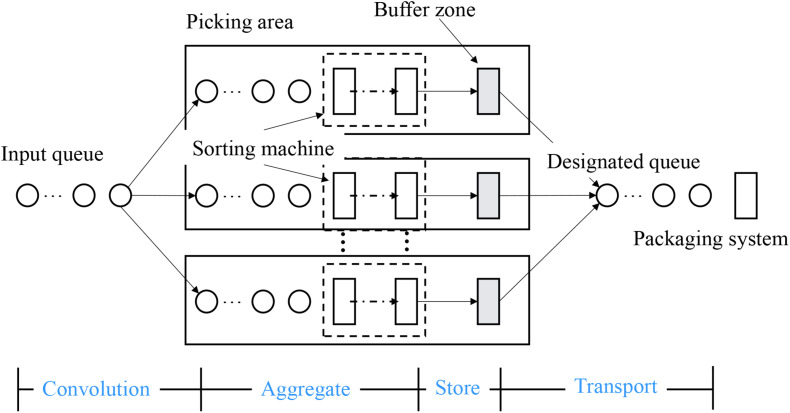
Distributed system for aggregation timing sequence.

The core of the RNN model lies in the ring structure on the left side, which expands the network in the timing direction and can learn the sequential relationship between the input samples by itself. In RNN, the input *X* of each hidden layer meets the following conditions:

(1) $$\Phi \left(Y\right)=\sum_{i}{\left(\sum_{j}w_{ij}X_{j}-X_{i}\right)}^{2}$$

(2) $$x_{i}^{k+1}=\sum_{j}w_{ij}x_{j}^{k}$$

(3) $$x_{i}^{k+1}=\frac{2}{D_{s}}\sum_{j}w_{ij}x_{j}^{k}$$

where *x*_*j*_ represents an element of *X*_*j*_, *w*_*ij*_ represents the weight between *x*_*i*_ and *x*_*j*_. It can be seen in Figure 5 that the network can theoretically look forward to any number of input nodes, but in the actual deep learning project, the basic cyclic neural network model cannot well deal with long input sequences. The main reason is that in the backpropagation training process of the RNN network loop layer, the longer the input sequence, the faster the error increases or decreases, and the corresponding problems such as gradient explosion or gradient disappearance lead to the network cannot be trained. To solve this problem, a large number of scholars have improved the basic RNN model, among which the most successful one is the long-term and short-term memory network model (LSTM), which was proposed by Hochreiter and Schmidhuber in 1997. It not only perfectly solves the problem of long-term dependence, but also improves the speech recognition performance of Google by nearly 50%.

## Data and Methods

### Data

The EEG signals used in this subject are from the BCI competition in 2005 and the other part is collected by the laboratory itself. The selection of subjects will have a certain impact on the universality and reliability of the experimental results. The EEG signals used in this study come from 2 women and 3 men, respectively. The subjects are all healthy, mental, and right-handed students. According to the previous physiological knowledge of cerebral cortical division, we can know that the cerebral cortex activated by imagining hand movement is larger and easier to detect, so this experiment stipulates that the subjects’ imagination task is left-hand finger movement and right-hand finger movement. Because of the characteristics of low signal-to-noise ratio, strong randomness, some matters must be paid attention to in the process of EEG acquisition.

**FIGURE 6:**
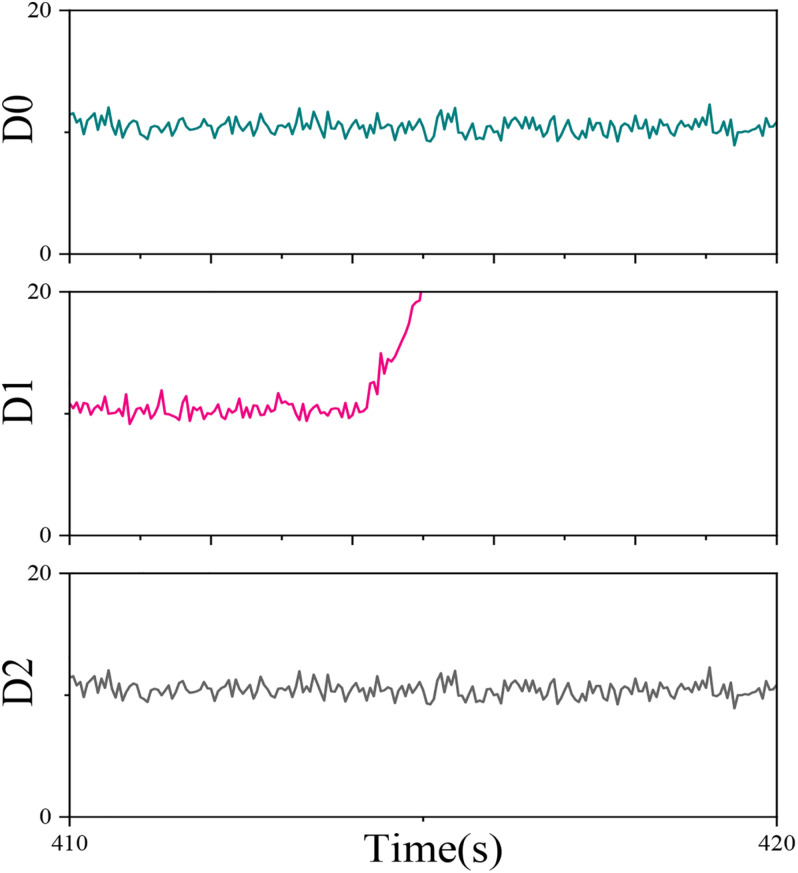
Communication module for different sensors.

Electroencephalogram signals are affected by blood glucose factors, so subjects are not recommended to conduct experiments on an empty stomach or in a full stomach, as shown in Figure 6. The subjects should wash their hair in advance. Too much scalp oil will lead to excessive scalp resistance and distortion of the acquisition waveform. EEG signals are extremely weak and easy to interfere with, so the process of data acquisition should be far away from electromagnetic interference and ensure a quiet collection environment. Internal factors can also be the interference source of EEG signals, so subjects should not carry out any exercise in the process of collection, keep muscles relaxed and minimize blinking, eye movement, saliva swallowing and other behaviors, to reduce the interference of EMG and ophthalmogram. The acquisition process of BCI competition is described as follows: subjects sit quietly in front of a computer wearing an electrode cap and imagine the movement of their left hand, right hand or right foot according to the on-screen graphics. Each subject had a total of 280 exercise imaginations, of which 140 were left and right.

In the experiment, the EEG voltage of the scalp was collected with a 118 conductive electrode cap. The position distribution of the 118 conductive electrodes in the cerebral cortex was shown above. The acquisition frequency was 100 Hz. The EEG signals of all the above processes were collected by the system, so the obtained EEG signals were five matrices with 118 rows 280 mm 7 columns, among which the effective EEG signals of each subject for motor imagination task were 118 rows 280 mm 3.5 column matrices. The EEG data collected by the laboratory use different acquisition equipment, and the rest of the process is similar to the above description, but only imagine the movement of the left and right fingers and use fixed arrows to minimize the eye movement interference of the subjects. The equipment used in the laboratory conducts electricity as much as 32 conductors, and the acquisition frequency is 250 Hz. Although the acquisition equipment is different, the principles of data preprocessing, classifier design and training are all the same. Therefore, in the latter description, all of them take BCI competition’s 118derivative data set as an example to explain the preprocessing and classifier network structure, and the operation process of 32-lead data is similar.

### Methods

#### Physiological Basis of Brain–Computer Interface

The structure of the human brain can be divided into left and right hemispheres, and each hemisphere can be divided into four lobes according to its location, namely frontal lobe, temporal lobe, parietal lobe, and occipital lobe. The lobar region contains nerve centers that can undertake different tasks, thus forming a phenomenon of zoning specialization in the cerebral cortex.

**FIGURE 7:**
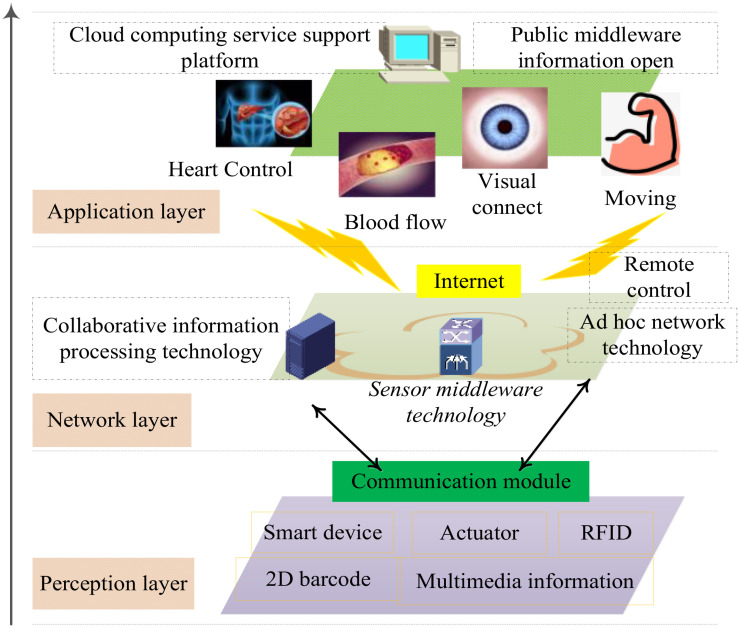
Distribution 0–2 (D0, D1, D2) changes with time.

In the different regions of the cerebral cortex, the somatic motor area and somatosensory area are closely related to motor imagination as shown in Figure 7. The somatosensory area is located in the frontal lobe near the central sulcus, which is mainly used to control the movement of the contralateral body. The somatosensory area is located in the parietal lobe near the central sulcus, which is mainly used to receive the sensation of the contralateral body. Although the subjects do not have real body movement in the process of motor imagination, the phenomena and characteristics that occur in these two regions are the same. There are mainly the following three points: (1) The two regions control and feel the contralateral body, that is, the somatomotor area of the left brain controls the movement of the right half of the body, and the somatosensory area of the left brain feels the sensation of the right half of the body, and vice versa. (2) The two areas control and feel the inverted body, that is, the upper part of the body motor area controls the movement of the lower limbs, the middle controls the movement of the upper limbs, the lower part controls the movement of the head, and so does the somatosensory area. (3) The area of different parts of the body mapped to the two areas is inversely proportional to the degree of flexibility and sensitivity of the part. For example, the hand and mouth can complete very complex movements, and their flexibility is much higher than that of the torso. Therefore, their area in the body movement area will be much larger than that of the torso.

There are more than 100 billion neurons in the human brain. When humans are thinking, these neurons will produce synchronous discharges. These potential changes can be collected by placing electrodes on the scalp, which is called EEG signals. The signal can be regarded as the superposition of waves with different frequencies, amplitudes, and phases produced by a large number of neurons. Brain waves can usually be divided into δ waves, θ waves, α waves, β waves, and γ waves. Because the EEG generated by motor imagination belongs to spontaneous EEG, and the frequency of spontaneous EEG is generally between 1 Hz and 30 Hz, so the γ wave can be filtered out directly without considering γ wave in BCI research. Among the five kinds of brain waves mentioned above, the α wave and β wave are closely related to the study of BCI. Specifically, they are the alpha wave in the middle-frequency band (mu rhythm) and the β wave that appears near the central sulcus. The frequency of mu rhythm is about 10 Hz, if classified according to frequency and amplitude, it can be classified as α wave, but mu rhythm has nothing to do with the state of eye closure, it is a kind of EEG signal which is closely related to somatic motor area. B waves are distributed near the frontal lobe and central sulcus, but the β waves related to mu rhythm often appear near the central sulcus (Mudiganty et al., 2017; Torres-Rodríguez et al., 2018; van Yperen et al., 2018). Therefore, in the study of BCI, we usually focus on the β waves near the central sulcus.

#### Motion-Dependent Synchronization Phenomenon

Event-related synchronous (ERS) / desynchronized (ERD) phenomenon is an electrophysiological phenomenon that can be used as a feature of motor imagination EEG signals. The specific manifestation of ERD phenomenon is as follows: when the unilateral limb is imagined or the real movement occurs, the somatic motor area and somatosensory area of the contralateral cerebral cortex will be activated, and the increase of blood flow and the acceleration of metabolism in this area will occur the decrease of α wave frequency and amplitude (α wave blocking phenomenon) (Belash et al., 2018). Correspondingly, the ERS phenomenon is shown as follows: because the somatic motor area and somatosensory area of the ipsilateral cerebral cortex are not activated, the frequency and amplitude of α wave will increase (Maceira-Elvira et al., 2019). ERD and ERS phenomena are closely related and can influence each other. Studies have shown that once the ERD phenomenon begins to occur in a certain region, it is accompanied by the ERS phenomenon in the adjacent cortex, and with time, the ERD phenomenon will gradually spread to the somatosensory motor areas on both sides. It shows the ERD/ERS phenomenon. C3 is the guide electrode placed in the somatic motor area of the left hemisphere, and C4 is the guide electrode in the somatic motor area of the right hemisphere. It can be seen that during the left-handed motor imagination task, the energy of alpha wave (12 Hz) in the somatic motor area (C4) of the right hemisphere decreased significantly. Also, this phenomenon can be calculated by the quantitative formula (2−1) (Telles et al., 2019), where *E* is the energy value of the α-wave band after motion imagination, and *R* is the energy value of the α-wave band before motion imagination. If ERSERD0/occurs, the ERD phenomenon occurs, otherwise, the ERS phenomenon occurs.

## Results and Discussion

In this section, we will report our experimental settings and results. Our experiments are conducted on a PC with Intel i7-9700 CPU @3.00 GHz, 32G memory, and RTX 1080ti 11G. All parameters in our method are determined by 5-CV.

### Rehabilitation Treatment of Motor Dysfunction

The EEG signals in the previous section are classified according to different motor imagination tasks, in which there are 5140,700 samples of imagining left finger movement and imagining right finger movement, and each sample is a matrix with a dimension. First of all, we make an intuitive time-domain analysis of the EEG signal, and in this process, by observing the average voltage of each guide electrode in different experiments, we can roughly judge the effectiveness of the EEG signal. If there is an obvious difference in amplitude or frequency between the EEG data collected in a certain experiment and the EEG data in most experiments, it can be considered that the EEG data is abnormal in the process of collection and is an invalid sample. After the abnormal data are put forward through macroscopic analysis, the specific electrodes can be further analyzed. The C3 conductive pole located in the somatic motor area of the left hemisphere is taken as an example. The primitive EEG signals collected during left-hand movement imagination and right-hand movement imagination are shown below. It can be seen that the event-related synchronization phenomenon (ERS), with the increase of α-wave amplitude at the (CLASS1) C3 electrode during the left-hand motion imagination and the event-related desynchronization phenomenon (ERD), with the decrease of the α-wave amplitude at the (CLASS2) C3 electrode during the right-hand motion imagination, are consistent with the physiological phenomena introduced before.

Then the EEG signal is analyzed in the frequency domain, and the periodic graph method is used to estimate the power spectrum. The Fourier transform of discrete-time series [*x* (1), *x* (2), …, *x* (*n*)] is calculated as equation (4), and the power spectral density function *P* (*w*) is shown as equation (5). When the signal sequence is of finite length, the expectation and de-limit operation of the omission formula (6) get the periodic graph estimation.

(4) $$P\left(w\right)=\sum_{i}{\left(\sum_{j}w_{ij}X_{1}-X_{2}\right)}^{2}$$

(5) $$F\left(w\right)=\sum_{i}q\left(n\right)e^{-jwn}$$

(6) $$P\left(w\right)=\sum_{i}{\left({\frac{1}{N}}_{i}\left(x_{1}-x_{2}\right)\right)}^{2}$$

**FIGURE 8:**
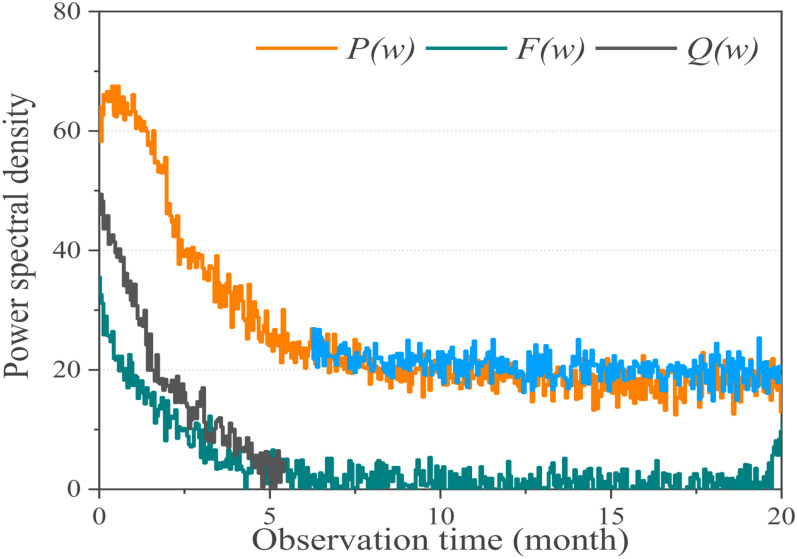
Power spectral density for *P*(*w*), *F*(*w*), and *Q*(*w*).

The power spectral density obtained from the power spectrum estimation of the 118-channel EEG signal is shown in Figure 8. From this image, we can directly see the distribution of EEG signals on 0–50 Hz, but the result is contrary to the theory in above section. In theory, the delta wave at 0–4 Hz is only visible in deep sleep, while the delta wave shown in the picture accounts for a large proportion in the EEG, even exceeding the alpha wave in the 8–14 Hz band as a feature. Thus it can be seen that a large number of low-frequency noise and 50 Hz power frequency interference in the original EEG data collected cannot be directly used for subsequent feature extraction and classification, so we should first carry out pre-processing operations such as noise reduction or reconstruction.

**FIGURE 9:**
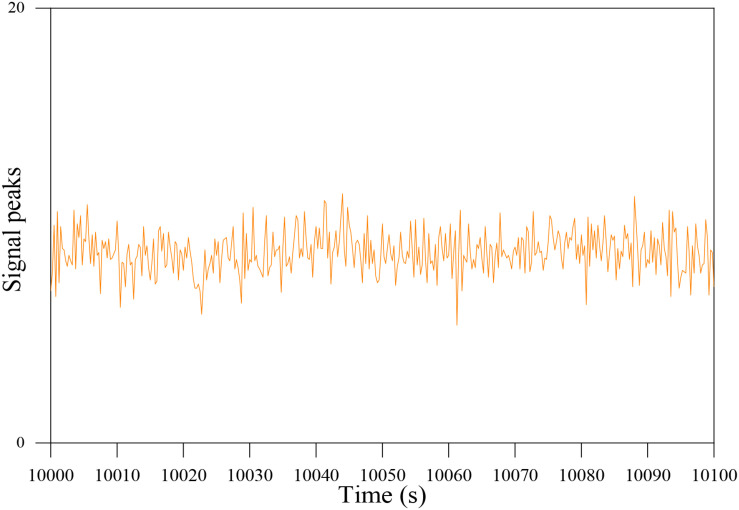
The signal peaks occur after 10000 s.

In this experiment, the sampling frequency of the EEG signal is 100 Hz. According to the Nyquist theorem, the maximum frequency retained in this signal is 50 Hz. Combined with the classification of 2.1.2 EEG signals according to frequency, the useful information in EEG signals is mainly concentrated in the middle and low-frequency band of 6.5 Hz–25 Hz. The frequency above 25 Hz can be regarded as high-frequency noise, and the frequency below 6.5 Hz has little to do with motor imagination. Therefore, the subject uses the fast algorithm of orthogonal wavelet transform Mallat algorithm for multi-resolution analysis of EEG signals, this algorithm only decomposes the low-frequency part of each layer, and the high resolution in the low-frequency band meets the needs of EEG band classification. After a large number of experiments, the subject uses the db6 function of the Daubechies wavelet system to decompose the original EEG signals in three layers, and the high-frequency and low-frequency parts of each layer are segmented as shown in above section, namely EEG = D1+D2+D3+A3. In the process of EEG reconstruction, only the two main frequency bands, D2 in the second layer and D3 in the third layer, are retained, and the wavelet coefficients of A3 in the low-frequency band and D1 in the first layer are set to 0 to achieve the purpose of noise reduction and enhancement of the signal, as shown in Figure 9. Taking the EEG signal collected by the C3 guide electrode during left-hand motion imagination as an example, wavelet analysis is carried out. The decomposed high-frequency coefficient d1murd3 and low-frequency coefficient A1-A3 results are shown above. The delta wave and theta wave between 0 Hz and 6.5 Hz, the α wave between 6.5 Hz and 12.5 Hz, the β wave between 12.5 Hz and 25 Hz, and the γ wave above 25 Hz are reconstructed by wavelet coefficients as shown below.

**FIGURE 10:**
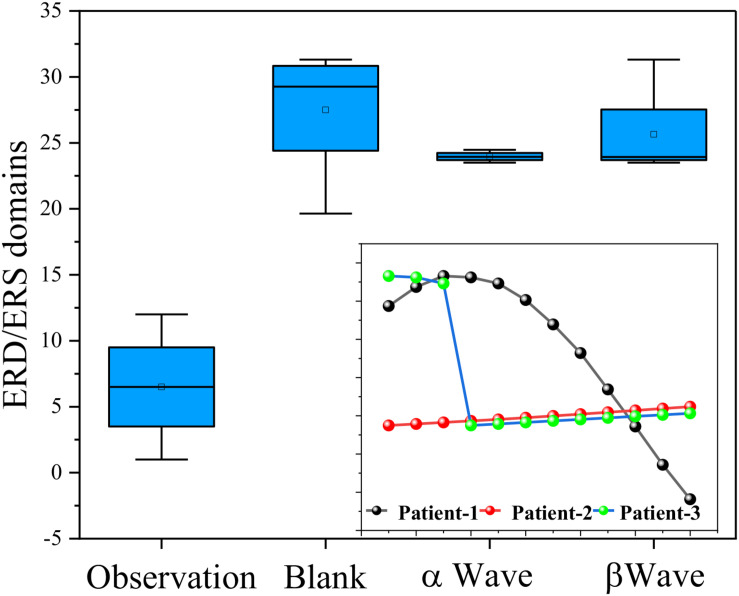
The ERD/ERS domains for α wave and β wave.

The above α-wave and β-wave are selected for reconstruction, and the EEG signal with the main frequency band of 6.5–25 Hz is obtained. The EEG signal after the above preprocessing operation is in the frequency domain, and the time domain analysis chart is shown. It can be seen from the picture that the signal is indeed consistent with the prior knowledge in brain theory. There is an obvious ERD/ERS phenomenon in time domain analysis, and the main components in frequency domain analysis are α wave and β wave as shown in Figure 10. Therefore, compared with the original data, the EEG preprocessed by wavelet analysis reduces the noise and enhances the features, and can be used for subsequent feature extraction and classification.

### Evaluation of Rehabilitation Effect

**FIGURE 11:**
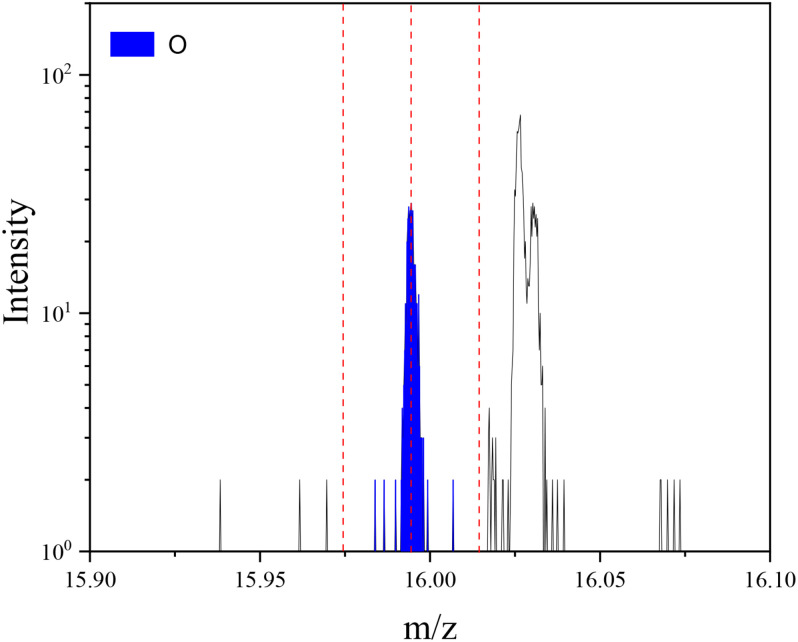
The MRI signal for limb stroke rehabilitation system.

Through functional electrical stimulation of muscles that are out of nerve control, muscles can contract and can replace or correct the lost motor functions of organs and limbs. Because the nervous system has a certain ability of self-repair and reorganization, according to the principle of human neurophysiology, electrical stimulation of nerves and muscles is used to stimulate afferent nerves and train movement information repeatedly. It will be introduced into the central nervous system, which can form exciting traces in the cerebral cortex and play a great role in promoting the recovery of lost motor function. Limb stroke rehabilitation system is generally divided into upper limb rehabilitation and lower limb rehabilitation, which can be detected by MRI mapping as shown in Figure 11. It is very important for the functional rehabilitation of the upper limb, especially for the wrist to complete the activities of the daily life of stroke patients. Therefore, in our experiment, the repeated stimulation of the upper limb is mainly aimed at the wrist flexion and wrist extension, while the lower limb is mainly aimed at the ankle dorsiflexion and metatarsal flexion.

**FIGURE 12:**
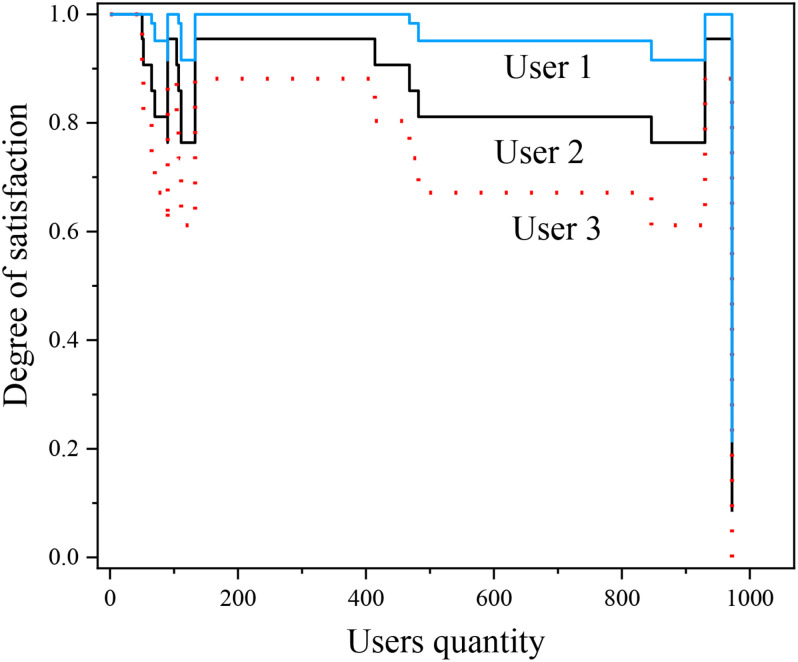
The degree of satisfaction for different users.

Brain–computer interface technology is a research field that has attracted wide attention and developed rapidly in the past 15 years. With the application of brain–computer interface technology, without relying on the transmission of intermediate neurons, a reliable information transmission channel can be established directly between the brain and external devices, to realize the direct control of EEG information to external devices. By decoding the electrical activity of cortical neurons, brain–computer interface technology provides a new interactive way for people with dyskinesia to acquire the motor ability and environmental control ability. Functional electrical stimulation has been widely used in the field of stroke rehabilitation and has been paid attention to by the majority of rehabilitation workers. Proper FES can cause the contraction of the corresponding muscles, so it can compensate for the lost limb movement. At the same time, the degree of satisfaction for different users is also uploaded to the afferent nerve and finally mapped to the advanced nerve center, which promotes the reconstruction of limb motor function as shown in Figure 12. This article makes an exploratory study on the design of a stroke rehabilitation system with the combination of brain–computer interface and functional electrical stimulation, which can show the feasibility of its realization. The EEG signals used in this subject are from the BCI competition in 2005 and the other part is collected by the laboratory itself. The selection of subjects will have a certain impact on the universality and reliability of the experimental results. The EEG signals used in this study come from 2 women and 3 men, respectively. The subjects are all healthy, mental, and right-handed students.

**FIGURE 13:**
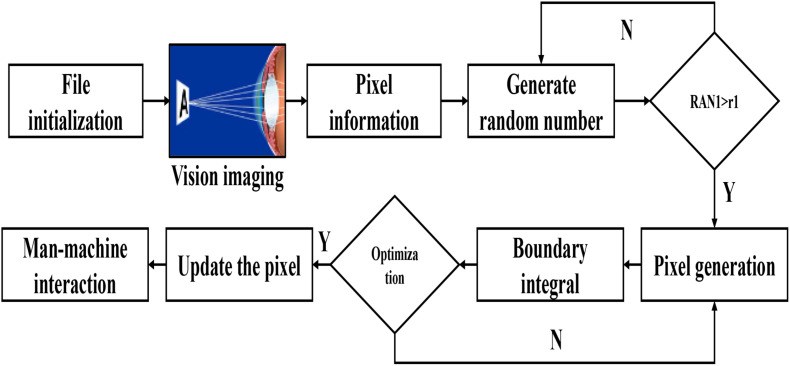
The vision imaging interacted with pixel generation.

Usually, for stroke hemiplegic patients, they lose the ability of spontaneous movement because the motor nerve cannot receive motor signals, but the muscles they dominate still have the ability of motor contraction, and the upper motor neurons of hemiplegic patients are damaged, losing the ability to control random movement and showing spastic paralysis, but the lower motor neurons are normal, so the pathway exists and has stress function. At this time, appropriate FES can contract the corresponding vision imaging and compensate for the loss of limb motor function as shown in Figure 13. At the same time, the stimulation is also uploaded to the afferent nerve and mapped to the higher nerve center through the spinal cord, which promotes the reconstruction of limb function and the recovery of mental state. Too much scalp oil will lead to excessive scalp resistance and distortion of the acquisition waveform. EEG signals are extremely weak and easy to interfere with, so the process of data acquisition should be far away from electromagnetic interference and ensure a quiet collection environment. Internal factors can also be the interference source of EEG signals, so subjects should not carry out any exercise in the process of collection, keep muscles relaxed and minimize blinking, eye movement, saliva swallowing and other behaviors, to reduce the interference of EMG and ophthalmogram. The acquisition process of BCI competition is described as follows: subjects sit quietly in front of a computer wearing an electrode cap and imagine the movement of their left hand, right hand or right foot according to the on-screen graphics. Each subject had a total of 280 exercise imaginations, of which 140 were left and right.

FES can be used for central paralyzes, such as hemiplegia, cerebral palsy, paraplegia, multiple lateral sclerosis of the spinal cord, and paralysis caused by spinal cord injury. It can also be used to relieve pain, treat epilepsy, spinal deformity, urinary incontinence, respiratory dysfunction, but also has the effect of visual aid, hearing aid, and even can be used in midwifery in the process of delivery. For the poor performance of LSTM classifier in the classification of motor imagination EEG signals, one of the reasons for the low accuracy of the test set may be that the experiment did not design a correct network, or did not effectively preprocess the data, wrong preprocessing methods, an insufficient amount of data and so on. Another reason may be that the characteristics of motor imagination EEG itself have little to do with time. Although the imaginative process of the subjects was sequential, the original principle of EEG classification by imagining left-handed and right-handed movements was: changes in the energy distribution and intensity of brain waves caused by the ERD/ERS phenomenon in the process of imagination.

(7) $$ERD/ERS=\frac{E-R}{R}\times 100\%$$

**FIGURE 14:**
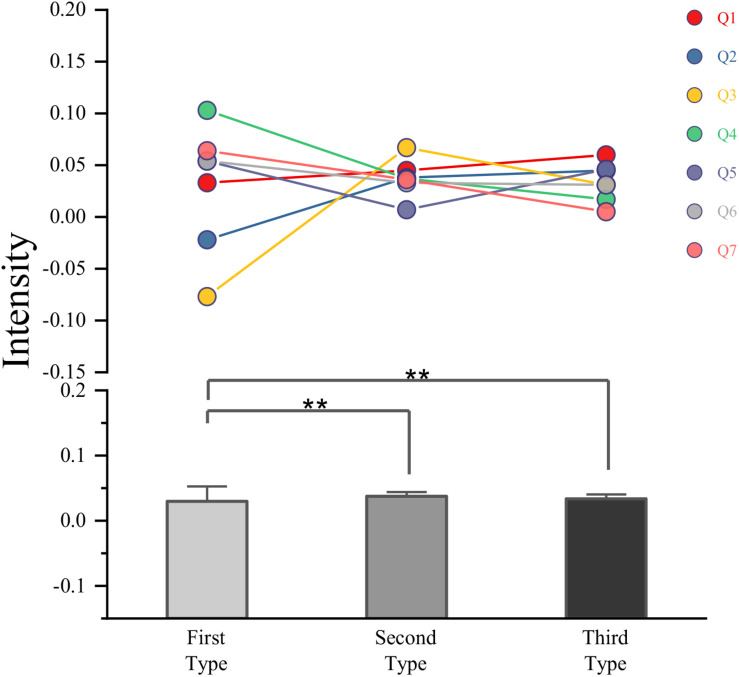
The signal intensity for different types.

That is to say when you imagine the movement of the right hand, the energy in the left brain region increases, and the energy in the right brain region decreases, while when you imagine the left-hand movement, it is just the opposite. These two features are mainly frequency domain features and spatial domain features, while the time domain features are relatively weak, so the classification effect of time series features learned by the LSTM algorithm in the training process is not as obvious as that learned by CNN algorithm. For the phenomenon that the classification accuracy of LSTM classifier when subjects are trained separately is not significantly higher than that when all subjects are trained together, but the accuracy of CNN classifier is quite different under these two methods, this article speculates that the frequency and spatial characteristics of EEG signals may be different because different subjects have different brain shapes and different ways of thinking. There are great differences in many types, such as different active parts of the brain, that is, the characteristics of EEG signals extracted by CNN of single-person network vary from person to person, while the time sequence characteristics of EEG signals are relatively fixed and are not affected by the above-mentioned factors, and the difference may be small among different signal types. The signal intensity for different types can be seen in Figure 14. For convolution neural network, the data of different subjects can be trained together because the essence of deep learning is to find commonness in massive data to fit it, so even if there are individual differences in the characteristics of motion imagination among different subjects, the CNN of the multi-person network can also find commonness and extract more universal ERD/ERS features.

## Conclusion

Electroencephalogram has the characteristics of low signal-to-noise ratio, vulnerable to interference, and obvious differences between different individuals, which makes it difficult for traditional classification methods to find good differentiation and representative characteristics to design a classification model with excellent performance. With the above characteristics, brain–computer interface technology is expected to solve the physiological and psychological needs of patients with motor dysfunction with great individual differences. However, the classification method based on feature extraction requires a lot of prior knowledge when extracting data features and lacks a good measurement standard, which makes the development of brain–computer interface technology. In particular, the development of a multi-classification brain–computer interface is facing a bottleneck. However, in recent years, with the characteristics of layer-by-layer automatic learning data features, step-by-step abstraction, and good generalization ability, deep learning has achieved great success in the field of image and speech. To avoid the blindness and complexity of EEG feature extraction, the deep learning method is applied to the automatic feature extraction of EEG signals. It is necessary to design a classification model with strong robustness and high accuracy for EEG signals. Based on the research and implementation of a brain–computer interface system based on a convolutional neural network, this article aims to design a brain–computer interface system that can automatically extract features of EEG signals and classify EEG signals accurately. Avoid the blindness and time-consuming problems caused by the machine learning method based on feature extraction in feature extraction of EEG data due to the lack of a large amount of prior knowledge.

This study has several limitations. For example, overfitting often occurs during the classification procedure. Therefore, in our future work, we will try to overcome this problem to improve the generalization ability of our model.

## Data Availability Statement

All datasets presented in this study are included in the article/supplementary material.

## Author Contributions

HW designed the whole algorithm and experiments. ZY, FL, QZ, ZL, and FZ contributed to the code implementation. QS supervised the whole including experiments and manuscript writing. All authors contributed to the article and approved the submitted version.

## Conflict of Interest

The authors declare that the research was conducted in the absence of any commercial or financial relationships that could be construed as a potential conflict of interest.

## References

[B1] Arndt S. Laszig R. Aschendorff A. Hassepass F. Beck R. Wesarg T. (2017). Cochlear implant treatment of patients with single-sided deafness or asymmetric hearing loss. *Hno* 65 98–108. 10.1007/s00106-016-0297-5 28188428

[B2] Bai Z. Fong K. N. K. (2020). Remind-to-move treatment enhanced activation of the primary motor cortex in patients with stroke. *Brain Topogr.* 33 275–283. 10.1007/s10548-020-00756-7 32056031

[B3] Barfod K. W. Hansen M. S. Hölmich P. Kristensen M. T. Troelsen A. (2020). Efficacy of early controlled motion of the ankle compared with immobilisation in non-operative treatment of patients with an acute achilles tendon rupture: an assessor-blinded, randomised controlled trial. *Br. J. Sports Med.* 54 719–724. 10.1136/bjsports-2019-100709 31597624

[B4] Belash V. O. Mokhov D. E. Tregubova E. S. (2018). The use of the osteopathic correction for the combined treatment and rehabilitation of the patients presenting with the vertebral artery syndrome. *Voprosy Kurortologii Fizioterapii I Lechebnoi Fizicheskoi Kultury* 95 34–43. 10.17116/kurort20189506134 30499484

[B5] Bober S. L. Fine E. Recklitis C. J. (2020). Sexual health and rehabilitation after ovarian suppression treatment (Share-Os): a clinical intervention for young breast cancer survivors. *J. Cancer Survivorsh.* 14 26–30. 10.1007/s11764-019-00800-x 31482477

[B6] Bober S. L. Recklitis C. J. Michaud A. L. Wright A. A. (2018). Improvement in sexual function after ovarian cancer: effects of sexual therapy and rehabilitation after treatment for ovarian Cancer. *Cancer* 124 176–182. 10.1002/cncr.30976 28881456 PMC5734953

[B7] Dong V. A. Fong K. N. K. Chen Y.-F. Tseng S. S. Wong L. M. (2017). ‘Remind-to-Move’ treatment versus constraint-induced movement therapy for children with hemiplegic cerebral palsy: a randomized controlled trial. *Dev. Med. Child Neurol.* 59 160–167. 10.1111/dmcn.13216 27503605

[B8] Drew B. T. Conaghan P. G. Smith T. O. Selfe J. Redmond A. C. (2017). The effect of targeted treatment on people with patellofemoral pain: a pragmatic, randomised controlled feasibility study. *BMC Musculoskelet. Disord.* 18:338. 10.1186/s12891-017-1698-7 28778218 PMC5545020

[B9] Earnshaw V. A. Bogart L. M. Menino D. D. Kelly J. F. Chaudoir S. R. Reed N. M. (2019). Disclosure, stigma, and social support among young people receiving treatment for substance use disorders and their caregivers: a qualitative analysis. *Int. J. Ment. Health Addict.* 17 11–15. 10.1007/s11469-018-9930-8 PMC7731618 33312084

[B10] Ferragut-Garcías A. Plaza-Manzano G. Rodríguez-Blanco C. Velasco-Roldán O. Pecos-Martín D. Oliva-Pascual-Vaca J. (2017). Effectiveness of a treatment involving soft tissue techniques and/or neural mobilization techniques in the management of tension-type headache: a randomized controlled trial. *Arch. Phys. Med. Rehabil.* 98 211–219. 10.1016/j.apmr.2016.08.466 27623523

[B11] Jiang Y. Wu D. Deng Z. Qian P. Wang J. Wang G. (2017). Seizure classification from EEG signals using transfer learning, semi-supervised learning and TSK fuzzy system. *IEEE Trans. Neural Syst. Rehabil. Eng.* 25 2270–2284. 10.1109/tnsre.2017.2748388 28880184

[B12] Kim W. S. Lee K. Kim S. Cho S. Pailk N.-J. (2019). Transcranial direct current stimulation for the treatment of motor impairment following traumatic brain injury. *J. Neuroeng. Rehabil.* 16 34–45. 30683136 10.1186/s12984-019-0489-9 PMC6347832

[B13] Kubota S. Abe T. Kadone H. Shimizu Y. Funayama T. Watanabe H. (2019). Hybrid assistive limb (Hal) treatment for patients with severe thoracic myelopathy due to ossification of the posterior longitudinal ligament (Opel) in the postoperative acute/subacute phase: a clinical trial. *J. Spinal Cord Med.* 42 517–525. 10.1080/10790268.2018.1525975 30335588 PMC6718179

[B14] Levy J. Molteni F. Cannaviello G. Lansaman T. Roche N. Bensmail D. (2019). Does botulinum toxin treatment improve upper limb active function. *Ann. Phys. Rehabil. Med.* 62 234–240. 10.1016/j.rehab.2018.05.1320 29960017

[B15] Lopes A. Maló P. Nobre M. D. A. Sánchez-Fernández E. Gravito I. (2017). The Nobelguide§All-on-4§treatment concept for rehabilitation of edentulous jaws: a retrospective report on the 7-Years clinical and 5-years radiographic outcomes. *Clin. Implant Dent. Relat. Res.* 19 233–244. 10.1111/cid.12456 27758069

[B16] Maceira-Elvira P. Popa T. Schmid A.-C. Hummel F. C. (2019). Wearable technology in stroke rehabilitation: towards improved diagnosis and treatment of upper-limb motor impairment. *J. Neuroeng. Rehabil.* 16 8–18. 31744553 10.1186/s12984-019-0612-y PMC6862815

[B17] Mudiganty S. Daolagupu A. K. Sipani A. K. Das S. K. Alam J. E. Plumari S. (2017). Treatment of infected non-unions with segmental defects with a rail fixation system. *Strategies Trauma Limb Reconstr.* 12 45–51. 10.1007/s11751-017-0278-6 28236034 PMC5360676

[B18] Pavlova E. L. Lindberg P. Khan A. J. O’Donovan A. Neylan T. C. Gross J. J. (2017). Transcranial direct current stimulation combined with visuo-motor training as treatment for chronic stroke patients. *Restorat.Neurol. Neurosci.* 35 307–317. 10.3233/rnn-160706 28506002

[B19] Pereira M. G. Lynch B. Hall-Faul Pedras M. Quality S. (2019). of Life of women with urinary incontinence in rehabilitation treatment. *J. Health Psychol.* 24 254–263. 10.1177/1359105316650615 27302604

[B20] Pin-Yeh F. Qiang W. (2017). Effects of intensive exercise training combined with prophylactic antidepressant treatment on motor function and depression in patients with stroke. *Int. J. Sci.* 6 81–89. 10.18483/ijsci.1215

[B21] Polkey M. I. Qiu Z.-H. Zhou L. Zhu M.-D. Wu Y.-X. Chen Y.-Y. (2018). Tai chi and pulmonary rehabilitation compared for treatment-naive patients with copd: a randomized controlled Trial. *Chest* 153 1116–1124. 10.1016/j.chest.2018.01.053 29625777

[B22] Schneider K. J. Leddy J. J. Guskiewicz K. M. Seifert T. McCrea M. Silverberg N. D. (2017). Rest and treatment/rehabilitation following sport-related concussion: a systematic review. *Br. J. Sports Med.* 51 930–934. 10.1136/bjsports-2016-097475 28341726

[B23] Shulman J. Conroy C. Cybulski A. Smith K. R. Jervis K. Johnson H. (2020). Does intensive interdisciplinary pain treatment improve pediatric headache-related disability? *Disabil. Rehabi.* 14–28. 10.1080/09638288.2020.1762125 32406759

[B24] Skolasky R. L. Wegener S. T. Aaron R. V. Ephraim P. Brennan G. Greene T. (2020). The optimize study: protocol of a pragmatic sequential multiple assessment randomized trial of nonpharmacologic treatment for chronic, nonspecific low back pain. *BMC Musculoskelet. Disord.* 21:293. 10.1186/s12891-020-03324-z 32393216 PMC7216637

[B25] Smith M. M. F. Collins N. J. Mellor R. Grimaldi A. Elliott J. Hoggarth M. (2020). Foot exercise plus education versus wait and see for the treatment of plantar heel pain (Feet Trial): a protocol for a feasibility study. *J. Foot Ankle Res.* 13 34–56. 32384905 10.1186/s13047-020-00384-1 PMC7206811

[B26] Stan J. H. V. Dijkers M. P. Whyte J. Hart T. Turkstra L. S. Zanca J. M. (2019). The rehabilitation treatment specification system: implications for improvements in research design, reporting, replication, and synthesis. *Archiv. Phys. Med. Rehabil.* 100 146–155. 10.1016/j.apmr.2018.09.112 30267666 PMC6452635

[B27] Telles S. Sayal N. Nacht C. Chopra A. Patel K. Wnuk A. (2019). Yoga: can it be integrated with treatment of neuropathic pain? *Ann. Neurosci.* 26 82–91. 10.5214/ans.0972.7531.260208 31975778 PMC6894618

[B28] Tian X. Gao H. Jiang W. Zhang H. Yu X. Liu E. (2019). Deep multi-view feature learning for eeg-based epileptic seizure detection. *IEEE Trans. Neural Syst. Rehabil. Eng.* 27 1962–1972. 10.1109/tnsre.2019.2940485 31514144

[B29] Torres-Rodríguez A. Griffiths M. D. Carbonell X. (2018). The treatment of internet gaming disorder: a brief overview of the pipatic program. *Int.J. Ment. Health Addict.* 16 1000–1015. 10.1007/s11469-017-9825-0 30147635 PMC6096606

[B30] Turgut N. Möller L. Dengler K. Steinberg K. Sprenger A. Eling P. (2018). Adaptive cueing treatment of neglect in stroke patients leads to improvements in activities of daily living: a randomized controlled, crossover trial. *Neurorehabil. Neural Repair* 32 988–998. 10.1177/1545968318807054 30328767

[B31] van Yperen D. T. Reijman M. van Es E. M. Bierma-Zeinstra S. M. A. Meuffels D. E. (2018). Twenty-year follow-up study comparing operative versus nonoperative treatment of anterior cruciate ligament ruptures in high-level athletes. *Am. J. Sports Med.* 46 1129–1136. 10.1177/0363546517751683 29438635

[B32] Wang J. Shi W. Khiati D. Shi B. Chen M. Xia Z. (2020). Acupuncture treatment on the motor area of the scalp for motor dysfunction in children with cerebral palsy: study protocol for a multicenter randomized controlled trial. *Trials* 21 11–21. 31907027 10.1186/s13063-019-3986-z PMC6945653

[B33] Whyte J. Dijkers M. P. Hart T. Van Stan J. H. Packel A. Turkstra L. S. (2019). The importance of voluntary behavior in rehabilitation treatment and outcomes. *Archiv. Phys. Med. Rehabil.* 100 156–163. 10.1016/j.apmr.2018.09.111 30267665

[B34] Zesiewicz T. A. Wilmot G. Kuo S.-H. Perlman P. Greenstein P. E. Ying S. H. (2018). Comprehensive systematic review summary: treatment of cerebellar motor dysfunction and ataxia: report of the guideline development, dissemination, and implementation subcommittee of the american academy of neurology. *Neurology* 90 464–471. 10.1212/wnl.0000000000005055 29440566 PMC5863491

[B35] Zhang P. Hu S. He F. Fan J. Wang Q. He X. (2017). [He Xingwei’s exploration and experience in the pathogenesis and treatment of motor impairment of the trunk after stroke. *Chin. Acupunct. Moxibustion* 37 191–193. 10.13703/j.0255-2930.2017.02.024 29231485

[B36] Zhang Y. Chung F. Wang S. (2019a). A multiview and multiexemplar fuzzy clustering approach: theoretical analysis and experimental studies. *IEEE Trans. Fuzzy Syst.* 27 1543–1557. 10.1109/tfuzz.2018.2883022

[B37] Zhang Y. Chung F. Wang S. (2019b). Fast reduced set-based exemplar finding and cluster assignment. *IEEE Trans. Syst. ManCybernet.* 49 917–931. 10.1109/tsmc.2017.2689789

[B38] Zhang Y. Dong J. Zhu J. Wu C. (2019c). Common and special knowledge-driven TSK fuzzy system and its modeling and application for epileptic EEG signals recognition. *IEEE Access* 7 127600–127614. 10.1109/access.2019.2937657

[B39] Zhang Y. Li X. Zhu J. Wu C. Wu Q. (2019d). Epileptic EEG signals recognition using a deep view-reduction TSK Fuzzy system with high interpretability. *IEEE Access* 7 137344–137354. 10.1109/access.2019.2942641

[B40] Zhang Y. Chung F. Wang S. (2020a). Fast exemplar-based clustering by gravity enrichment between data objects. *IEEE Trans. Syst. Man Cybernet.* 50 2996–3009.

[B41] Zhang Y. Zhou Z. Bai H. Liu W. Wang L. (2020b). Seizure classification from EEG signals using an online selective transfer TSK fuzzy classifier with joint distribution adaption and manifold regularization. *Front. Neurosci.* 14:496. 10.3389/fnins.2020.00496 32595441 PMC7300255

[B42] Zhang Y. Ishibuchi H. Wang S. (2018). Deep Takagi–Sugeno–Kang fuzzy classifier with shared linguistic fuzzy rules. *IEEE Trans. Fuzzy Syst.* 26 1535–1549. 10.1109/tfuzz.2017.2729507

[B43] Zhang Y. Wang L. Wu H. Geng X. Yao D. Dong J. (2016). A clustering method based on fast exemplar finding and its application on brain magnetic resonance images segmentation. *J. Med. Imaging Health Informat.* 6 1337–1344. 10.1166/jmihi.2016.1923

[B44] Zhao K. Zhou L. Qian P. Ding Y. Jiang Y. Chen Y. (2019). A transfer fuzzy clustering and neural network based tissue segmentation method during PET/MR attenuation correction. *J. Med. Imaging Health Informat.* 9 1491–1497. 10.1166/jmihi.2019.2749

[B45] Zheng J. Cao J. Wang Z. Liu F. Wang S. Wu T. (2019). Semi-automatic synthetic computed tomography generation for abdomens using transfer learning and semi-supervised classification. *J. Med. Imaging Health Informat.* 9 1878–1886. 10.1166/jmihi.2019.2809

